# Multi-omics and machine learning identify novel biomarkers and therapeutic targets of COVID-19

**DOI:** 10.3389/fimmu.2025.1671936

**Published:** 2025-10-02

**Authors:** Yumei Zhou, Pengbei Fan, Haiyun Zhang, Shuai Han, Minghua Bai, Ji Wang, Qi Wang

**Affiliations:** ^1^ National Institute of Traditional Chinese Medicine (TCM) Constitution and Preventive Treatment of Disease, Wangqi Academy of Beijing University of Chinese Medicine, Beijing University of Chinese Medicine, Beijing, China; ^2^ School of Traditional Chinese Medicine, Southern Medical University, Guangzhou, China; ^3^ Medical Laboratory Center, Dalian Municipal Women and Children’s Medical Center (Group), Dalian, China; ^4^ Laboratory Animal Center of Inner Mongolia Medical University, Hohhot, China; ^5^ Hubei Shizhen Laboratory, Hubei University of Chinese Medicine, Wuhan, China

**Keywords:** multi-omics, scRNA-seq, RNA-seq, biomarkers, COVID-19

## Abstract

**Introduction:**

COVID-19 has caused over 7 million deaths worldwide since its onset in 2019, and the virus remains a significant health threat. Identifying sensitive and specific biomarkers, along with elucidating immune-mediated mechanisms, is essential for improving the diagnosis, treatment, and prevention of COVID-19. To predict key molecular markers of COVID-19 using an established multi-omics framework combined with machine learning models.

**Methods:**

We conducted an integrated analysis of single-cell RNA sequencing (scRNA-seq), bulk RNA sequencing, and proteomics data to identify critical biomarkers associated with COVID-19. The multi-omics approach enabled the characterization of gene expression dynamics and alterations in immune cell subsets in COVID-19 patients. Machine learning techniques and molecular docking analyses were employed to identify biomarkers and therapeutic targets within the disease’s pathophysiological network.

**Results:**

Principal component analysis effectively grouped samples based on clinical characteristics. Using random forest and SVM-RFE models, we identified clinical indicators capable of accurately distinguishing COVID-19 patients. Transcriptomic analysis, including scRNA-seq, highlighted the pivotal role of CD8^+^ T cells, and WGCNA identified related module genes. Proteomic analysis, integrated with machine learning, revealed 36 DEPs. Further investigation identified several genes associated with monocyte proportions. Correlation analysis showed that BTD, CFL1, PIGR, and SERPINA3 were strongly linked to CD8^+^ T cell abundance in COVID-19 patients. ROC curve analysis demonstrated that these genes could effectively distinguish between COVID-19 patients and healthy individuals. Concordant findings from both transcriptomic and proteomic levels support BTD, CFL1, PIGR, and SERPINA3 as potential auxiliary diagnostic markers. Finally, AlphaFold-based molecular docking analysis suggested these biomarkers may also serve as candidate therapeutic targets.

**Discussion:**

Preliminary findings indicate that BTD, CFL1, PIGR, and SERPINA3 are vital molecular biomarkers related of CD8+ T cell, providing new insights into the molecular mechanisms and long-term prevention of COVID-19.

## Graphical Abstract

Overview of the study design. The participants were divided into two groups: control group (n=265) and COVID-19 group (n=358). Principal component analysis was used to group samples according to clinical characteristics. Selected features were analyzed based on random forest model and SVM-REF model. By integrating scRNA-seq and RNA-seq data with an analysis of the peripheral plasma proteome, we applied machine learning models to successfully identify and predict potential biomarkers associated with CD8+ T cell responses in COVID-19 infection. ROC curve analysis was used to analyze the clinical diagnostic efficacy of inflammatory factors among different groups. The box diagram was used to show the levels of different inflammatory factors in plasma of different populations.

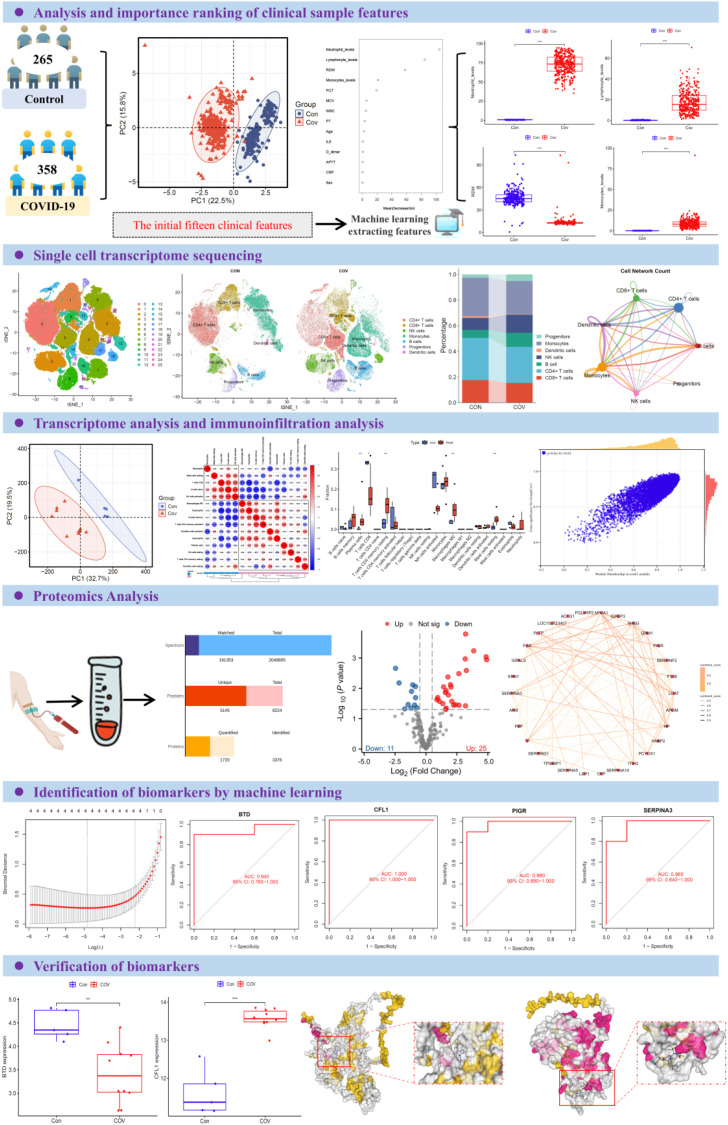

## Background

Despite a decline in the overall burden of COVID-19, the threat posed by the virus remains significant and persistent (1, 2). COVID-19, caused by the zoonotic SARS-CoV-2, continues to spread through respiratory droplets and direct contact, posing ongoing challenges to public health (3). Despite the flood of insights into the behavior of the virus and how to prevent it from causing harm (4, 5), many questions remain regarding the duration and quality of immunity after reinfection or vaccination, the impact of coinfections, and optimal treatment protocols for different populations. Severe COVID-19 is characterized by pneumonia, lymphopenia, exhausted lymphocytes and a cytokine storm (6). However, whether this is protective or pathogenic remains to be determined. Critical COVID-19-related illnesses continue to occur, and the virus’s impact on immune function remains insufficiently understood (7). Therefore, identifying sensitive and specific molecular markers, uncovering key immune mechanisms, and discovering new therapeutic targets are not only important but also critically necessary. Continued research is essential to manage COVID-19 effectively, mitigate its long-term effects, and prepare for future threats.

The integrated analysis of single cell transcriptome (scRNA-seq), bulk RNA-seq and proteomics has become a frontier means to analyze the mechanism of complex diseases and find accurate biomarkers (8). Single cell transcriptome technology can analyze the heterogeneity of cells with high resolution, and reveal the gene expression dynamics of specific cell subsets in microenvironment of COVID-19 patients (9, 10). However, the sparsity and high noise of single cell data need to be optimized by preprocessing and dimensionality reduction clustering, while the bulk transcriptome data provide the global expression characteristics of transcription level, and the two can be complemented by algorithms to infer the changes of cell subsets during the disease process (11). Proteomics data directly reflect the activity of functional molecules and make up for the lack of information in post-transcriptional regulation (12). Multimodality integration improves accuracy by combining single-cell data, bulk transcriptome, and serum proteomics, advancing early disease screening, target discovery, and personalized treatment in precision medicine.

COVID-19 infection can trigger an inappropriate immune response, leading to excessive activation of immune cells, tissue inflammation, and even multi-organ dysfunction (13). Current studies indicate that T cell-mediated immunity plays a crucial role in the effective antiviral response to COVID-19 (14). In this context, CD8+ T cells form an initial line of defense by rapidly recognizing viral antigens (15). COVID-19 infection induces CD8+ T cells to initiate cytotoxic responses, with cytotoxic T lymphocytes being responsible for clearing infected cells, thus playing a key role in controlling the virus (16, 17). Regulatory T cells also help prevent severe COVID-19 outcomes by mitigating excessive host inflammatory responses (18). In summary, cellular immunity, particularly CD8+ T cells, is vital for monitoring and predicting COVID-19 progression, aiding in early detection of severe cases and guiding clinical treatment. While patients with COVID-19 often present with lymphopenia, the disease has also been linked to immune hyperresponsiveness (19). Notably, the mechanisms behind immune cell dysfunction are not yet fully understood.

In this study, we performed integrated analyses using scRNA-seq, RNA-seq, proteomics, and AlphaFold-based molecular docking to investigate the alterations in key biomarkers associated with COVID-19. Functional enrichment analysis and machine learning were subsequently employed to identify pathogenic genes and elucidate their role within the pathophysiological network of COVID-19. By combining genetic evidence with clinical trial data, we developed a multi-omics framework to prioritize immune-mediated drug targets for COVID-19. Preliminary findings indicate that biotinidase (BTD), Cofilin 1 (CFL1), Polymeric Immunoglobulin Receptor (PIGR), and Alpha-1-antichymotrypsin (SERPINA3) are critical molecular markers associated with CD8+ T cells in the context of COVID-19, suggesting their potential as biomarkers for diagnosis and therapeutic intervention.

## Methods

### Clinical data collection

In this study, we retrospectively analyzed the test data of 358 COVID-19 patients and 265 healthy people who were treated in the Second Affiliated Hospital of Mudanjiang Medical College from January to June 2023. This study was conducted in strict accordance with the Declaration of Helsinki and was approved by the Medical Ethics Review Committee of the Second Affiliated Hospital of Mudanjiang Medical College (No. 202328). All participants gave their consent to participate in the study.

### Clinical features extract

Principal component analysis (PCA) was performed using the “prcomp” function from the “stats” R package based on the 14 clinical features. We utilized the randomForest v4.7 package to construct a random forest model for ranking the importance of these clinical features and evaluating their performance as indicators. Specifically, the number of decision trees (such as n tree ≥ 500), the maximum depth and other parameters are set, and the Gini index decline or replacement importance is used to evaluate the feature contribution.

### Single-cell RNA-seq

The scRNA-seq data used in this study were screened from the public database GEO database platform. The scRNA-seq dataset (GSE192391) used is based on the Illumina NovaSeq assay platform and contains 12 patients and 6 controls (10). Subsequent analysis was performed using the R Seurat v5.2.1 package. The following quality control steps were performed to filter the count matrices: Genes expressed in <3 cells and cells expressing fewer than 200 genes were removed; Cells expressing >5000 genes were discarded as these could be potential multiplet events where more than a single cell was encapsulated within the same barcoded GEM; Cells with >10% mitochondrial content was filtered out as these were deemed to be of low-quality. The data set consists of 18 samples. In order to remove the batch effect except the processing factors between different samples, data integration is carried out based on rPCA method. After data integration, the cells were clustered by principal component analysis and dimensionality reduction. According to the markers of human cell subsets determined in previous studies, cell annotation was carried out and the proportion of each cell subset was calculated. In this study, the R CellChat v1.6.1 package was used to infer the interaction between cells based on the expression of receptors and ligands on the cell surface. The algorithm simulates the probability of cell-to-cell communication by inputting the gene expression data of cells and combining the gene expression with the prior data of the interaction between signal ligands, receptors and their co-factors.

### DEG analysis, functional enrichment and immune infiltration analysis

The RNA-Seq data used in this study were GSE164805 included 10 COVID patients and 5 controls (20). Using R limma v3.62.2 package, according to the sample grouping, the differences between standard groups were analyzed. Cluster analysis was used to calculate PCA and FactoMine package drawing. Transcriptomics data were processed by R DESeq2 v1.46.0 package to analyze the difference of the original Counts matrix, and follow the standard process. In this study, |Log2FC| > 0.5 and *P* < 0.05 were set for differential expression genes filtration. Gene ontology (GO) enrichment analysis of DEGs was implemented in the R GO.db v3.20.0 packages based on Wallenius non-central hypergeometric distribution (https://geneontology.org/). The DEGs were analyzed using the Kyoto Encyclopedia of Genes and Genomes (KEGG) database (https://www.genome.jp/kegg/pathway.html). Protein-protein interaction network was conducted in STRING (https://string-db.org/). Transcriptome data was transformed into the total abundance of immune cells by utilizing the Cell-type Identification by Estimating Relative Subsets of protein (CIBERSORT) analysis with the R CIBERSORT v0.1.0 package. Using the Wilcoxon test, immune cells were compared among COVID samples and control samples.

### Construction of co-expression network

Weighted gene co-expression network analysis (WGCNA) was utilized to identify gene modules with similar expression patterns and analyze the correlation between monocyte proportion and gene modules. The scale independence and average connectivity of the networks were tested with different power values (from 1 to 20). The appropriate power value was determined when the independent scale was greater than 0.85 and the connectivity was high. Then, the similarity matrix was transformed into a topological matrix with the topological overlap measure (TOM) describing the correlation between genes. The genes were clustered by using 1-TOM as the distance. A dynamic hybrid cutting method was used to establish a hierarchical clustering tree to identify co-expressed gene modules. Each leaf of the tree represents a gene, and genes with similar expression data aggregate to form a branch of the tree and each branch represents a gene module. A weighted co-expression network model was established, and the gene expression matrix was divided into several related modules. Finally, the modules related to KOA and immune cell infiltration were selected for further analysis.

### Label-free protein profiling detection

Protein quantification was performed using the Bradford protein quantification kit (purchased from Shanghai Biotechnology Company). Trypsin (purchased from Solebac (Beijing)) was added to each protein sample and incubated at 37°C for 4 h. CaCl_2_ was added to each sample and digested overnight. Formic acid was added, centrifuged at 12,000 g for 5 min, and the resulting supernatant was loaded onto a C18 desalting column. LC-MS/MS analysis was performed using an EASY-nLC TM 1200 UHPLC and Q Exactive TM HF-X mass spectrometry system, both purchased from ThermoFisher (USA), operated in data-dependent acquisition (DDA) mode.

### DEP analysis, functional enrichment and PPI analysis

Proteomics data were processed by R DEP version 1.26.0 package. Proteomic data were then normalized using “normalize_vsn” function. Cluster analysis was used to calculate PCA and R package FactoMine drawing. “add_rejections” function was applied to calculate the fold-change values of proteins. In this study, |Log2FC| > 0.5 and *P* < 0.05 were set for differential expression proteins filtration. GO enrichment analysis of DEPs was implemented in the GOseq R packages based on Wallenius non-central hypergeometric distribution. The DEPs were analyzed using the KEGG database. Protein-protein interaction network was conducted in STRING.

### Machine learning techniques

Machine learning was employed as a tool to improve biomarker screening. First, feature selection was performed using the Random Forest (RF) algorithm, which calculates the mean decrease in Gini (MDG) for each gene or protein, ranks them accordingly, and determines the optimal number of features by sequentially adding differential genes/proteins from highest to lowest MDG until classification accuracy is maximized. Next, the least absolute shrinkage and selection operator (LASSO), implemented via the “glmnet” R package, was applied as a regularized regression approach to further refine the selected features. Finally, an artificial neural network (ANN) analysis was conducted with the R packages “neuralnet” for model construction and training, and “NeuralNetTools” for visualization and interpretation of the results. This integrated workflow enabled robust identification and evaluation of candidate biomarkers.

### ROC analysis and validation of the biomarkers

In addition, the expression level and diagnostic value of biomarkers was verified in both proteomics and transcriptomics data. We examined the diagnostic effectiveness of the biomarkers with the ROC using the R pROC v1.18.5 package. The expression levels of the biomarkers were also compared between COVID and control samples using an independent t-test, with *P* < 0.05 considered statistically significant.

### AlphaFold-based molecular docking

To assess the binding affinity between the COVID-19 antiviral drug Nirmatrelvir/Ritonavir (Paxlovid) (21) and key biomarkers, the 3D structures of core target proteins were predicted using AlphaFold DB (https://alphafold.ebi.ac.uk/), while the 3D structure of Paxlovid (PubChem CID, 155903259) was retrieved from PubChem (https://pubchem.ncbi.nlm.nih.gov/). Binding affinity was evaluated using CB-Dock2, with a docking score ≤ –5.0 kcal/mol considered indicative of strong interaction (22, 23).

### Statistical analysis

The normal distribution of the data was assessed by the Shapiro-Wilk normality test using or SPSS Statistics v27.0 (IBM, USA), and the mean is expressed as mean ± standard error. If the data were normally distributed, the differences between the means were assessed by one-way analysis of variance and Tukey’s multiple comparison test. If the data were not normally distributed, scores were compared using the nonparametric Kruskal-Walli’s test. When *p* < 0.05, the difference between groups was considered statistically significant. A professional biostatistician reviewed the study and confirmed that the sample size is appropriate and statistically justified.

## Result

### Analysis of clinical sample features

**Figure 1:**
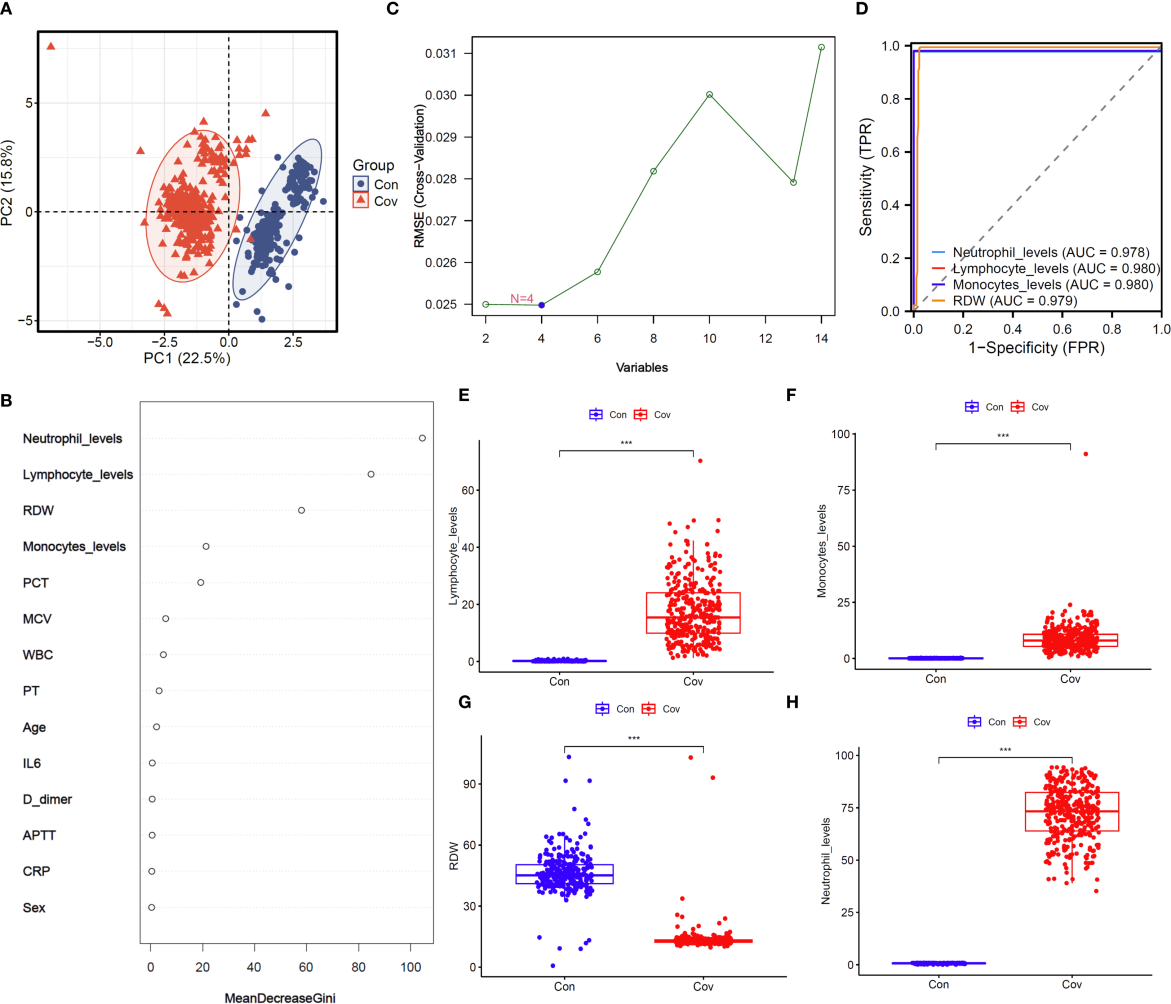
Clinical data collection and features extract. **(A)** PCA analysis. **(B)** Importance of clinical characteristic of random forest analysis. **(C)** Extraction of clinical features by SVM-REF. **(D)** ROC analysis of four clinical features. **(E)** Levels of lymphocytes in blood of two groups. **(F)** Levels of monocytes in blood of two groups. **(G)** RDW in blood of two groups. **(H)** Levels of neutrophil in blood of two groups.

A total of 623 samples were collected and divided into two groups: Control (n=265) and COVID-19 (n=358). PCA shows that the horizontal and vertical coordinates (PC 1 and PC 2) explain 22.5% and 15.8% of the total variation respectively, and the cumulative contribution rate is 38.3%, which indicates that it can effectively capture the main variation trend of samples data (**Figure 1A**, **Table 1**). The random forest results suggest that the top five clinical features of the five importance scores are lymphocyte level, monocyte level, red cell distribution width (RDW), neutrophil level and PCT (**Figure 1B**). The results of SVM-REF indicated that four clinical features changed obviously when the disease occurred, namely lymphocyte level, monocyte level, RDW and neutrophil level (**Figure 1C**). Moreover, the model exhibited favorable performance with ROC curve, lymphocyte level (AUC = 0.98), monocyte level (AUC = 0.98), RDW (AUC = 0.979) and neutrophil level (AUC = 0.978) (**Figure 1D**). To further screen the clinical sample features for machine learning model construction, we analyzed the changing trends of significant clinical sample features among different groups (**Figures 1E–H**).

**Table 1:** Clinical data analysis.

n	Control (265)	Covid (358)	*P value*
Age (mean (SD))	60.45 (19.18)	68.77 (14.39)	<0.001
WBC.Count (mean (SD))	7.71 (4.47)	6.98 (3.39)	<0.001
Neutrophil.Proportion (mean (SD))	66.07 (17.38)	70.83 (16.05)	<0.001
Lymphocyte.Proportion (mean (SD))	21.66 (15.42)	17.10 (10.45)	<0.001
Monocyte.Proportion (mean (SD))	8.53 (4.49)	8.54 (6.21)	0.054
RBC.Mean.Volume (mean (SD))	91.30 (8.20)	88.79 (14.70)	0.016
RBC.Distribution.Width (mean (SD))	14.19 (2.58)	12.70 (2.43)	<0.001
PT (mean (SD))	8.38 (6.48)	10.29 (5.07)	<0.001
APTT (mean (SD))	18.36 (13.79)	23.26 (11.43)	<0.001
D.Dimer (mean (SD))	0.99 (1.86)	0.96 (2.30)	<0.001
IL6 (mean (SD))	1.14 (5.63)	11.25 (31.03)	<0.001
PCT (mean (SD))	0.56 (3.97)	0.22 (1.13)	<0.001
C.Reactive.Protein (mean (SD))	42.41 (52.10)	33.68 (53.20)	<0.001
Sex = 2 (%)	119 (44.9)	151 (42.2)	<0.001

### scRNA-seq reveals the key immune cell components of COVID-19 patients

**Figure 2:**
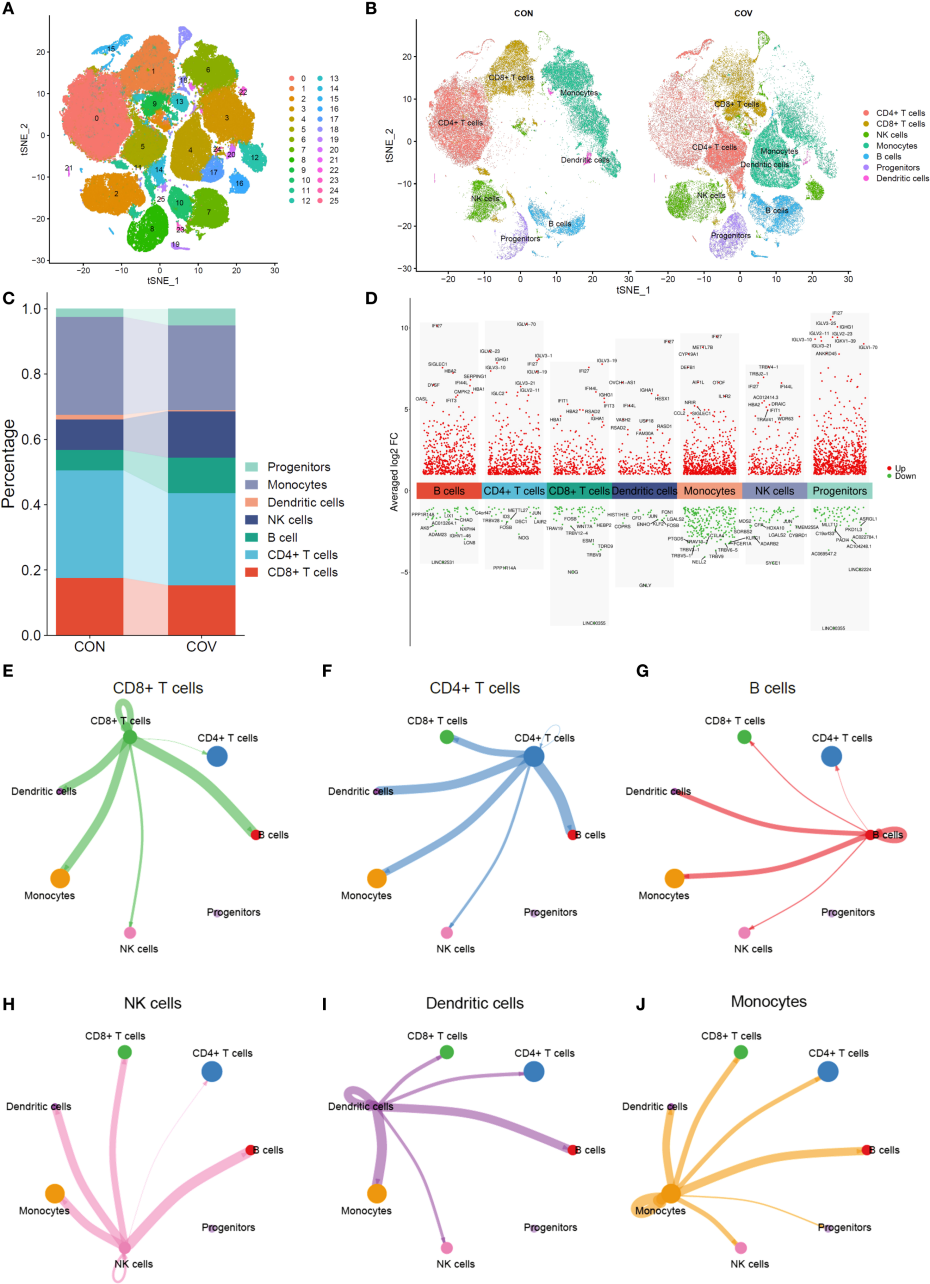
Single-cell RNA sequencing analysis. **(A)** t-SNE analysis of single-cell transcriptome. **(B)** Comparative t-SNE analysis between control and COVID-19 groups. **(C)** Cell Cluster Distribution in control and COVID-19 groups. **(D)** Differentially expressed genes (DEGs) across cell clusters **(E–J)**. Cell interaction network maps.

We compared the distribution characteristics of single cells between control group and COVID-19 group by ISNE dimensionality reduction technique. **Figure 2A** shows the distribution pattern of 26 cell subsets in a two-dimensional space composed of ISNE_1 (horizontal axis) and ISNE_2 (vertical axis) in the form of digital clustering, while the spatial location of CD8+ T cells (orange), monocytes (blue-green), dendritic cells (pink) and other major immune cells is clearly marked on the right (**Figure 2B**). Among them, the COVID-19 group showed remarkable characteristics: the density of CD8+ T cells in ISNE_1 axis 10–20 and ISNE_2 axis -10–0 regions increased obviously, showing antiviral immune activation; The distribution range of monocytes expanded to ISNE_1 axis 15–25 and ISNE_2 axis -5-5, and partially overlapped with T cell region, suggesting cell interaction in inflammatory microenvironment. Dendritic cells distributed discretely between 0–10 of ISNE_1 axis and 5–15 of ISNE_2 axis, which may be related to the disorder of antigen presentation. These differences in spatial distribution directly reveal the migration of immune cells, the reconstruction of subsets and the change of functional status caused by COVID-19 infection, which provides visual evidence for the analysis of virus-specific immune response. By comparing the immune cell composition and gene expression characteristics between the control group and COVID-19 group, the immune remodeling law of COVID-19 infection was revealed. The histogram of **Figure 2C** shows that the proportion of CD8+ T cells (red stripes) in COVID-19 group decreased significantly, while the proportion of monocytes (lavender stripes) increased relatively, suggesting that the proportion of immune cells was unbalanced. **Figure 2D** volcanic map further analyzed the functional changes, the cytotoxicity-related molecules (such as GZMB and IFNG) in CD8+ T cells gene expression profile were significantly increased (red dots were dense), indicating that the remaining cells were highly activated; Monocytes are accompanied by the strong expression of pro-inflammatory genes such as IL1β and TNF (the red dots in the fifth column on the right gather at a high level), which proves that they are polarized to the inflammatory phenotype. The up-regulation of CXCL9/10 chemokine in dendritic cells (red dot in the fourth column) and the fluctuation of NK cell activation gene (sixth column) jointly reveal the abnormal recruitment and regulation network disorder of immune cells in inflammatory microenvironment. It is worth noting that although the proportion of progenitor cells (light blue-green strips) is stable, their stem cell maintenance genes (such as SOX4) are down-regulated (the last green dot), suggesting that the immune regeneration potential is impaired. Then, we analyzed the cell interaction network formed a dynamic regulatory system through radial connection. Among them, CD8+ T cells (green center) establish multi-directional connections with CD4+ T cells (blue), dendritic cells (uncolored), B cells (red), monocytes (orange) and NK cells (pink) through green lines, highlighting its position as a hub of cytotoxic response (**Figure 2E**); CD4+ T cells (blue center) are connected in series with B cells (red thick junction), monocytes and progenitor cells through dense blue radiation, revealing their dual functions of coordinating humoral immunity and inflammatory regulation (**Figure 2F**). The strong connection between B cells (red center) and CD4+ T cells (red thick line) confirms the core pathway of antibody production in cooperation with T-B (**Figure 2G**), NK cells and T/monocytes form rapid killing defense through pink connection (**Figure 2H**), while dendritic cells are simultaneously connected with T/B/NK cells (**Figure 2I**) to mediate antigen presentation and chemotaxis recruitment. Monocytes (orange center) are widely connected with T/NK/dendritic cells through orange lines, suggesting that they play a bridging role in inflammatory signal amplification (**Figure 2J**). From the perspective of systems biology, we reveal the precise framework of cell cooperation in immune response.

### RNA-seq reveals the key immune components of COVID-19

**Figure 3:**
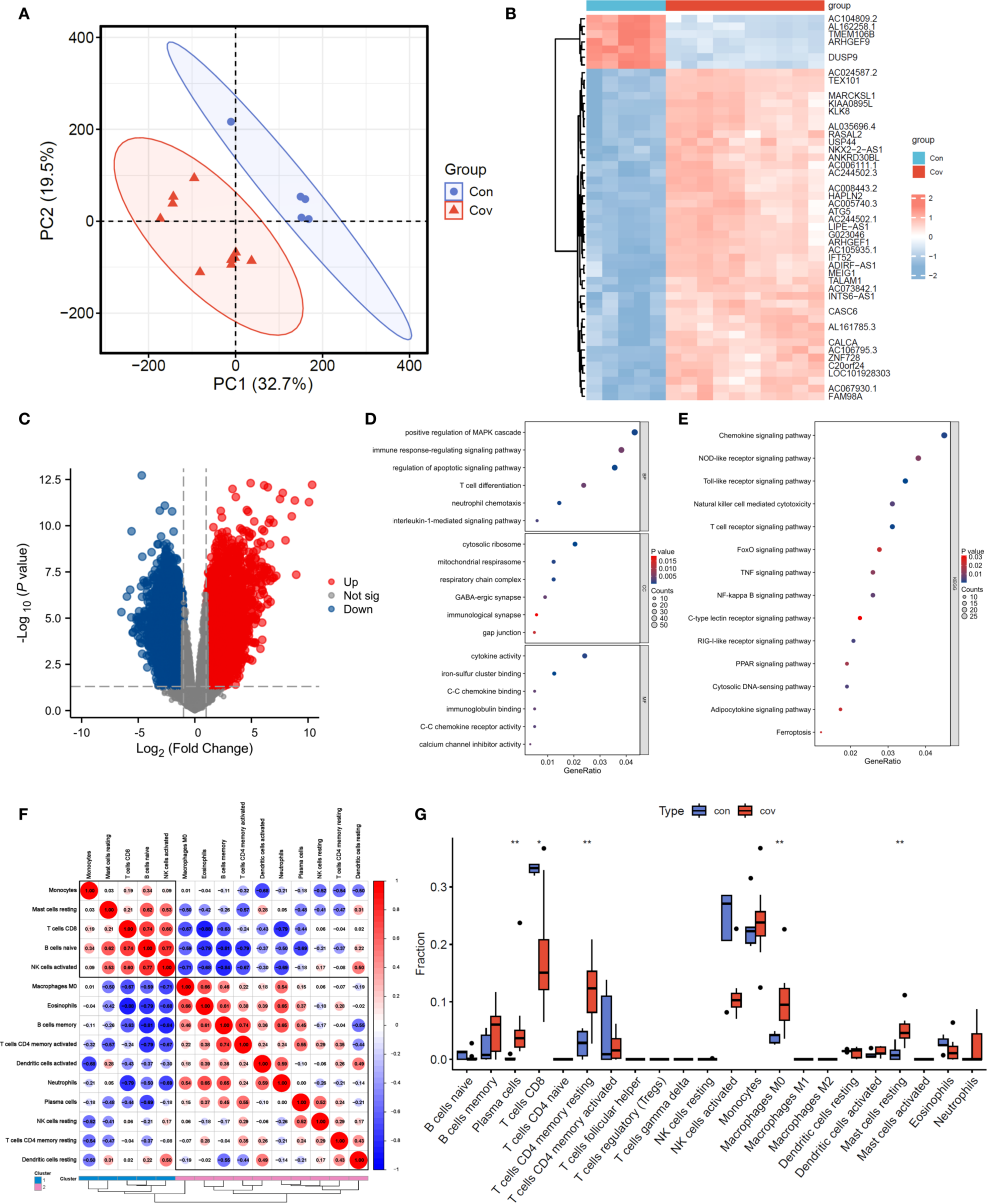
Transcriptomic data analysis. **(A)**. PCA analysis. **(B, C)**. Heatmap and volcano plot of transcriptomic data. **(D, E)**. GO and KEGG analyses. **(F, G)**. Heatmap and bar chart of immune infiltration analysis.

Through principal component analysis, the sequencing data of transcription group showed that the control group (Con, blue dot) and COVID-19 group (Cov, red triangle) were significantly separated along the PC1(32.7% variance) and PC2(19.5% variance) axes, among which the samples in the control group were relatively concentrated, suggesting that the transcription characteristics in the group were highly consistent (**Figure 3A**). Cluster thermogram shows the expression gradient of genes (rows) in different samples (columns), and blue to red respectively correspond to low expression to high expression level (**Figure 3B**). It can be seen that “Con” (blue label) and “Cov” (red label) samples form obvious clustering partitions on the gene expression spectrum. Further difference analysis showed that 3024 genes were up-regulated and 3812 genes were down-regulated (**Figure 3C**). GO analysis showed that the differential genes were significantly enriched in physiological process-related pathways such as MAPK cascade positive regulation, immune response regulation, apoptosis signal pathway and T cell differentiation, and involved ribosome structures, suggesting that cell function and structure were widely affected during the disease process (**Figure 3D**). KEGG enrichment results suggest innate immune pathways such as chemokines, Toll-like receptors and T cell receptors, as well as key regulatory hubs such as FoxO, TNF and NF-κB (**Figure 3E**).

The results of immune infiltration analysis showed that the correlation network among immune cell subsets was revealed by red and blue gradient (**Figure 3F**), in which CD8+ T cells were positively correlated with naive B Cell (r=0.74) and negatively correlated with neutrophils (r=-0.79), suggesting the cooperative or antagonistic relationship between different immune cells in the infiltration process. The difference in the proportion of immune cells between “con” (blue) and “cov” (red) groups was compared by box chart (**Figure 3G**). Among them, Plasma cells, CD4+ T cells memory resting, M0 macrophage, resting mast cells and CD8+ T cells showed significant distribution deviation between the two groups, and the difference in box span suggested that the treatment might specifically reshape the microenvironment of specific immune subgroups.

### WGCNA analysis reveals the modular genes of CD8+ T cells

**Figure 4:**
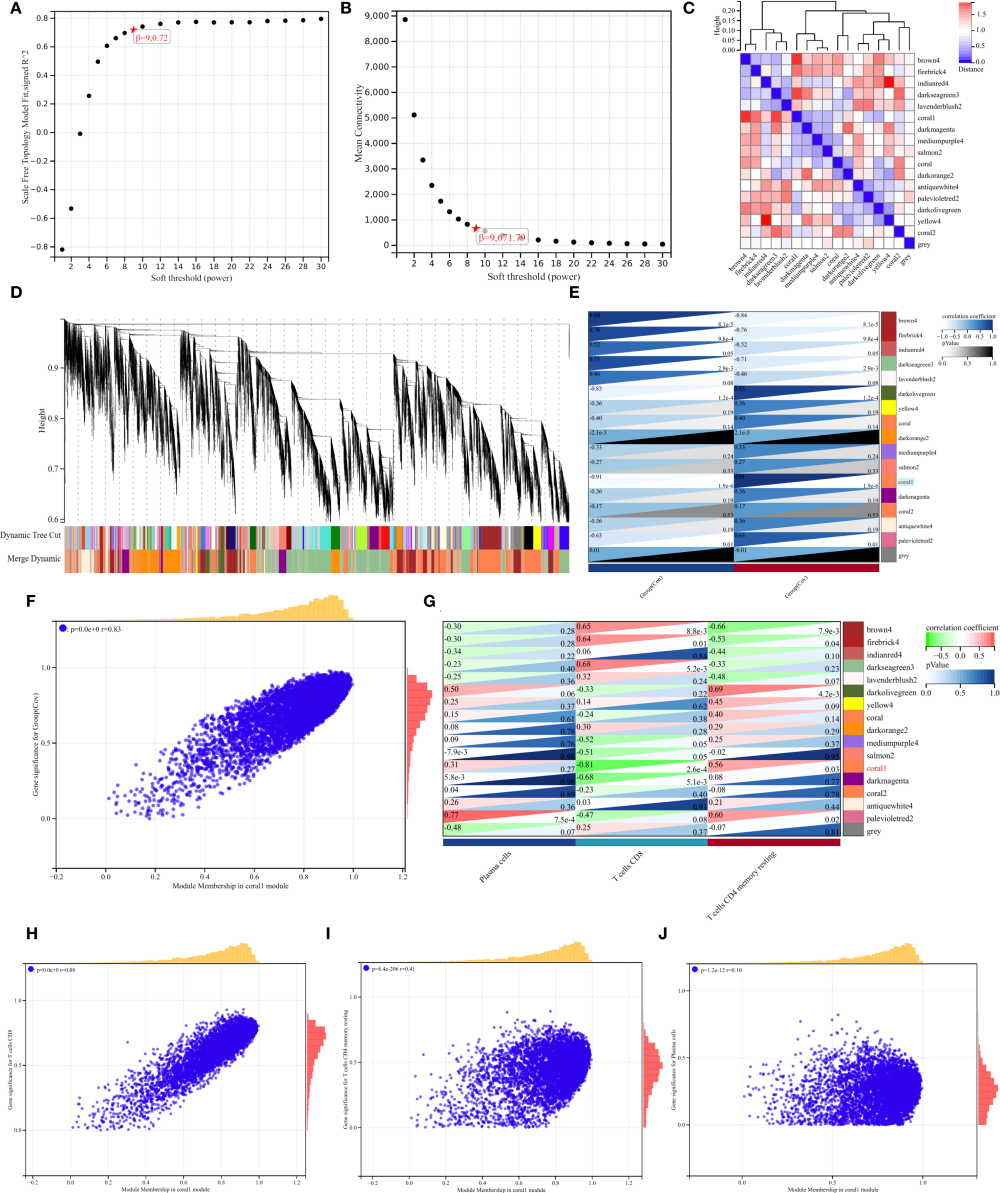
WGCNA Analysis. **(A, B)**. Selection of optimal soft thresholds for network construction. **(C)**. Heatmap of module-trait correlations. **(D)**. Hierarchical clustering dendrogram for module detection. **(E)**. Heatmap showing the correlation between modules and immune cells. **(F, G)**. **(H-J)** The correlation analysis between coral1 module gene and CD8+T cells **(H)**, CD4+T cells resting memory **(I)**, Plasma cells **(J)**.

**Figure 4A** shows the relationship between soft threshold (power) and goodness of fit (R^2^) of scale-free topological model. When power=9 (marked by red five-pointed star), R^2^ reaches 0.72 and the curve tends to be flat, indicating that this threshold can effectively balance the biological characteristics and topological attributes of the network. **Figure 4B** shows the decreasing trend of Mean Connectivity with the increase of power, and the connectivity drops to 671.79 when power=9, suggesting that the network retains the core co-expression structure after filtering weakly related genes. The thermogram of **Figure 4C** reveals the correlation pattern between modules. The dark red block in diagonal area (such as brown4 module) reflects the high degree of gene cooperation within modules, while the blue-red difference between firebrick4 and light-colored modules (such as r=0.32 to -0.45) implies the independence or antagonistic relationship of different functional modules. **Figure 4D** quantifies the similarity between samples/gene clusters by vertical height (vertical axis), and the black branching structure shows that low-level merged clusters (such as height < 5) have high homogeneity, while high-level branches (such as height > 15) suggest significant cross-cluster heterogeneity; At the bottom, “Dynamic Tree Cut” (color strip) marks the independent clusters (such as purple and orange clusters) generated by dynamic cutting, and “Merge Dynamic” strip shows the merged large-scale distribution (such as blue-green cross-sample continuous area), indicating that the data has multi-level clustering characteristics. Each module gene was correlated with the group (**Figure 4E**), and the results showed that coral1 module gene was significantly correlated with Cov group (r=0.91, *p* < 0.05). Correlation analysis of co-expression network showed that there was a strong positive correlation between the membership degree of coral1 module gene and the significance of weighted genes in Cov group (**Figure 4F**). By analyzing the interaction network between gene module and immune cell-related subsets, it was found that coral1 module gene was negatively correlated with CD8+ T cells (r=-0.81, *p* < 0.05), which emphasized the negative regulatory effect of Coral1 module gene on CD8+T cells (**Figure 4G**). Then, we analyzed the correlation analysis between coral1 module gene and CD8+T cells (**Figure 4H**, r=0.88), CD4+T cells resting memory (**Figure 4I**, r=0.41) and Plasma cells (**Figure 4J**, r=0.10).

### Proteomics analysis reveals the key proteins related with CD8+ T cells

**Figure 5:**
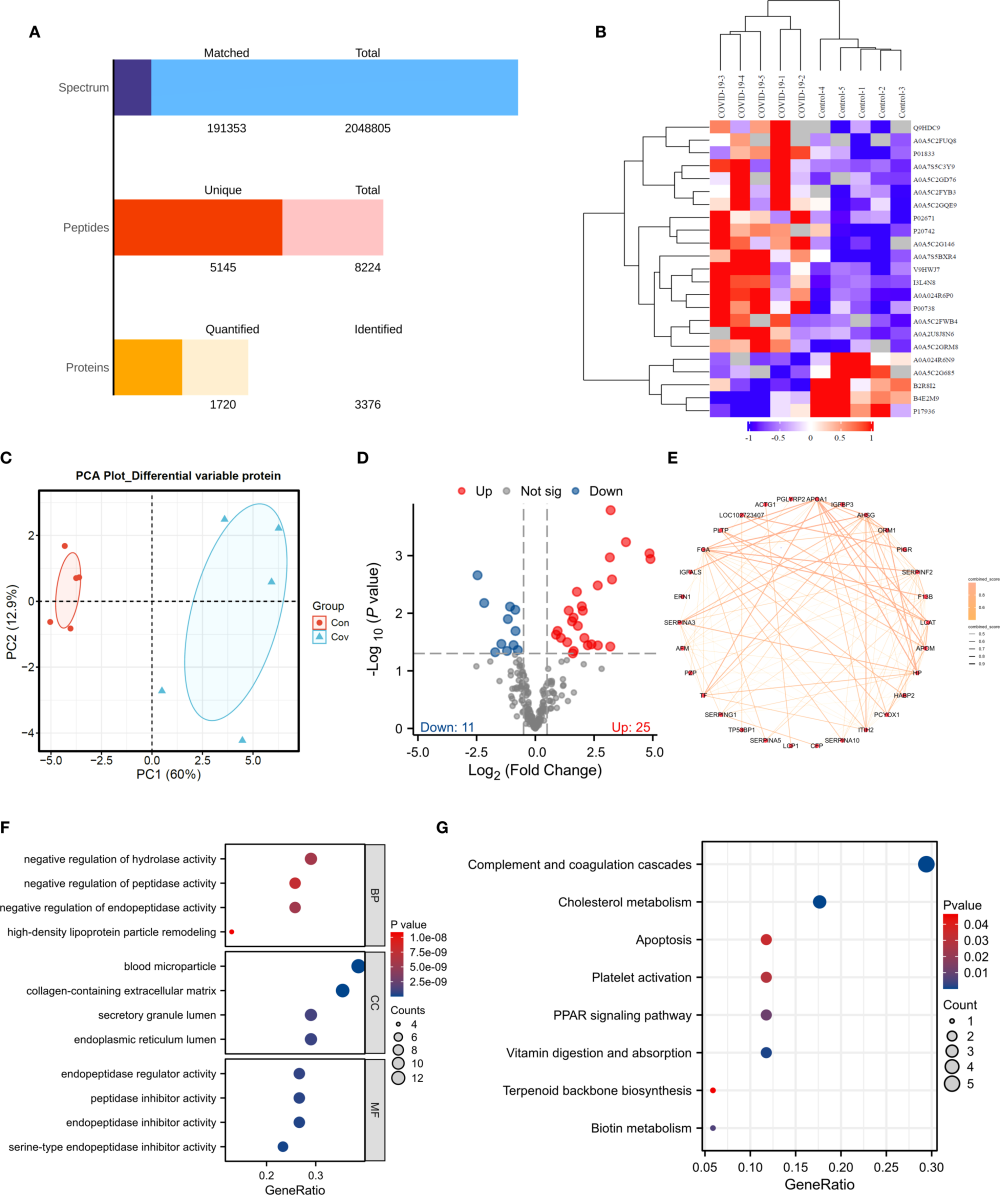
Proteomic Analysis. **(A)** Data quality assessment bar chart. **(B)** Clustering heatmap. **(C)** PCA analysis. **(D)** Volcano plot. **(E)** PPI analysis. **(F, G)**. GO and KEGG analyses.

The efficiency of proteomics analysis from original map to protein quantification (**Figure 5A**). In order to identify biomarkers of COVID-19, we conducted proteomic sequencing. The similarity between samples is shown in **Figure 5B**, where there is a significant difference between the control and COVID-19. PCA results demonstrate that COVID-19 has a difference compared to Control (**Figure 5C**). We found a total of 36 DEPs with 11 downregulated proteins and 25 upregulated proteins (**Figure 5D**). To further identify potential biomarkers, we conducted PPI network analysis using the DEPs (**Figure 5E**). To further understand the functions of these DEPs, we performed enrichment analysis. The results revealed that these differentially expressed genes are mainly enriched in biological processes such as negative regulation of hydrolase activity, negative regulation of peptidase activity, and negative regulation of endopeptidase activity (**Figure 5F**). They are also enriched in the apoptosis, PPAR signaling pathway and biotin metabolism (**Figure 5G**).

### Biomarker identification and molecular docking verification

**Figure 6:**
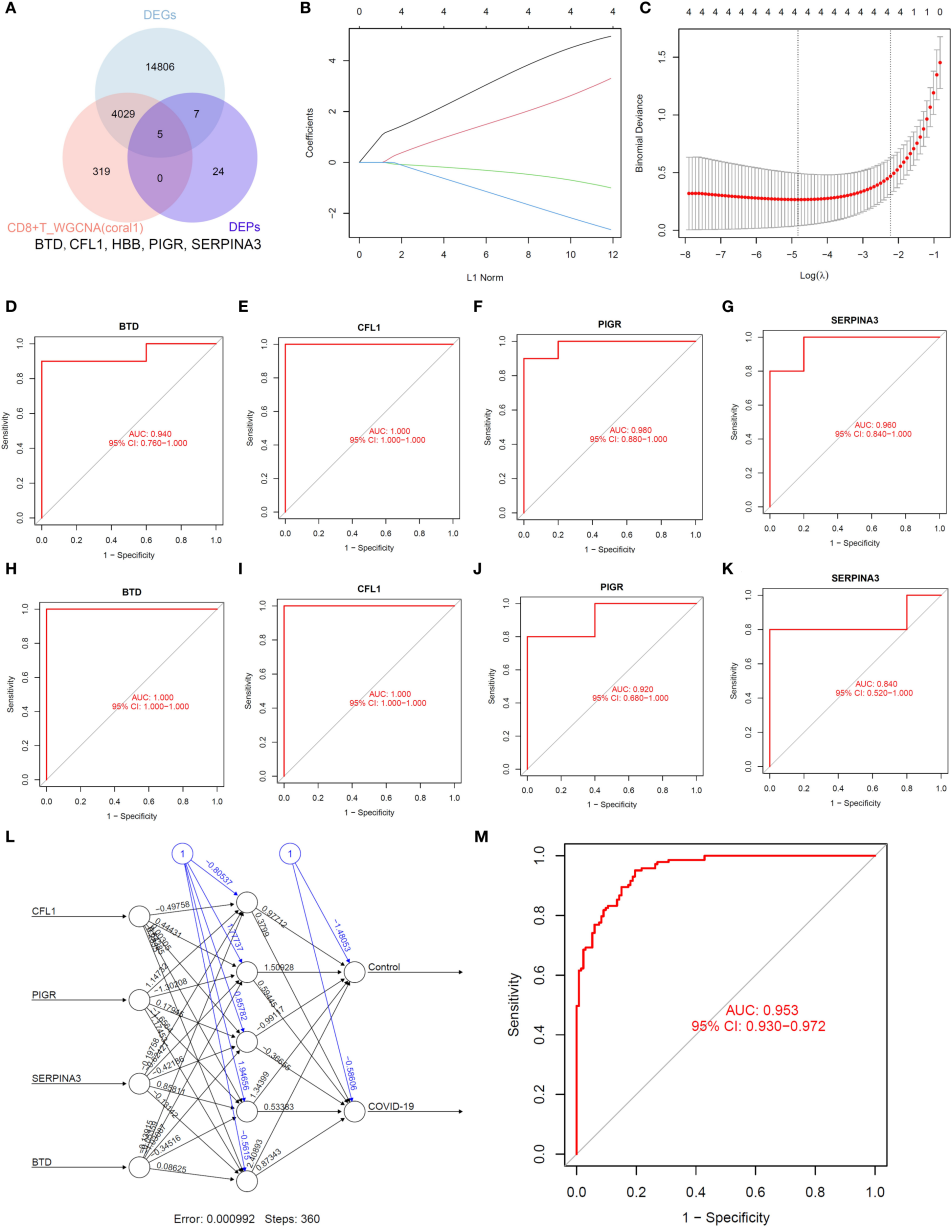
Identification of biomarkers. **(A)**. Venn diagram. **(B, C)**. LASSO regression analysis. **(D–G)**. ROC analysis of transcriptomics. **(H–K)**. ROC analysis of proteomics. **(L, M)**. ANN analysis.

Subsequently, we selected proteins from this module that intersected with differentially expressed proteins and hub genes (**Figure 6A**). We identified BTD, CFL1, PIGR and SERPINA3 as potential biomarkers with machine learning (**Figures 6B-C**). ROC analysis of transcriptomics results showed that BTD (AUC = 0.94, **Figure 6D**), CFL1 (AUC = 1, **Figure 6E**), PIGR (AUC = 0.98, **Figure 6F**), SERPINA3 (AUC = 0.96, **Figure 6G**) had high diagnostic value. ROC analysis of proteomics results showed that BTD (AUC = 1, **Figure 6H**), CFL1 (AUC = 1, **Figure 6I**), PIGR (AUC = 0.92, **Figure 6J**), SERPINA3 (AUC = 0.84, **Figure 6K**) had high diagnostic value. ANN model was constructed using four candidate biomarkers (CFL1, PIGR, SERPINA3, and BTD) as input variables (**Figure 6L**). The network comprised an input layer with four nodes, one hidden layer, and an output layer with two nodes corresponding to the Control and COVID-19 groups. The model converged with a final error of 0.000992 after 360 training steps. The diagnostic performance of the ANN was assessed by receiver operating characteristic (ROC) curve analysis. As shown in **Figure 6M**, the model achieved an area under the curve (AUC = 0.953), indicating excellent discriminative power in distinguishing COVID-19 patients from controls. These results suggest that the identified biomarkers, when integrated into an ANN framework, hold strong potential for accurate disease classification.

**Figure 7:**
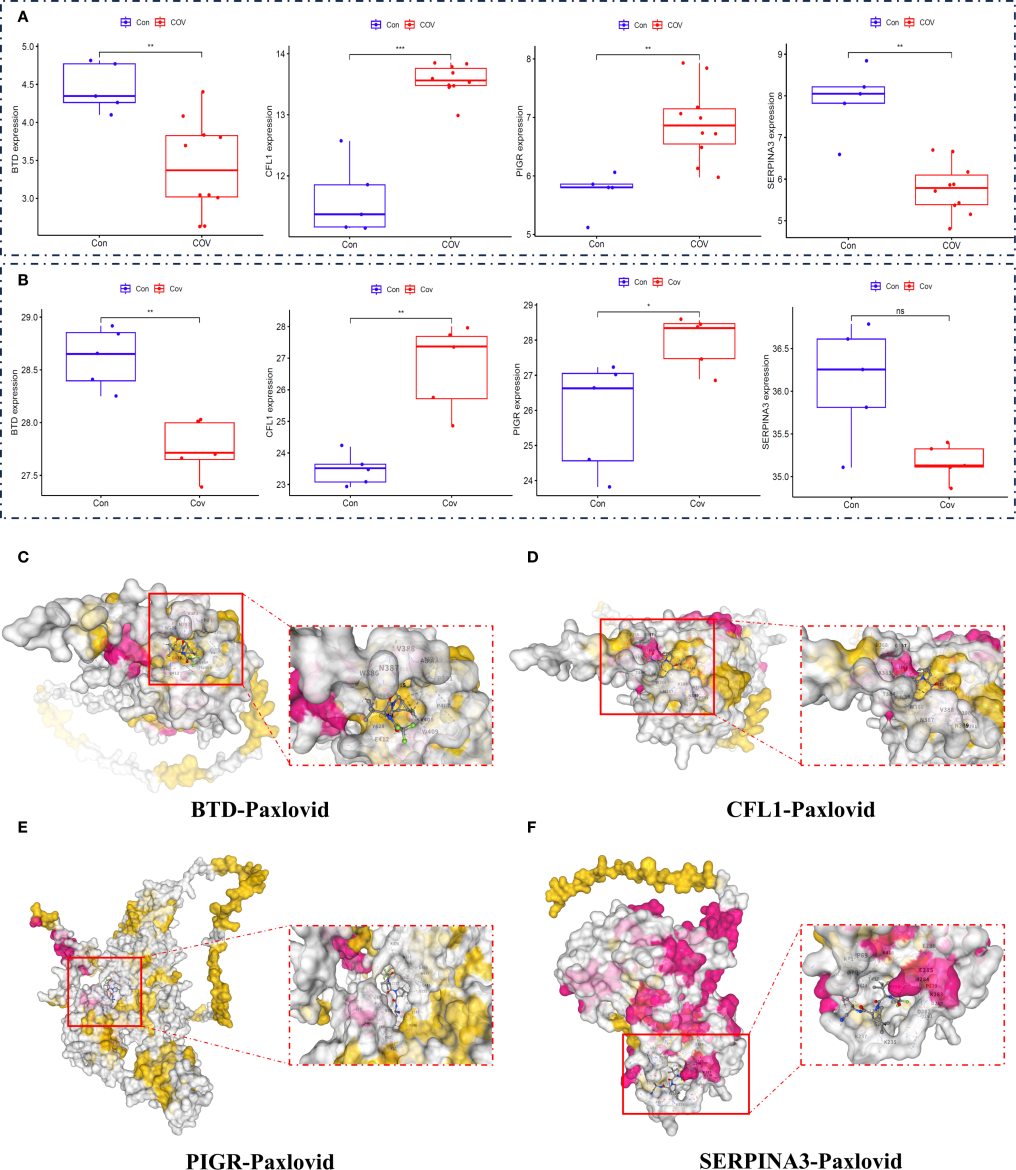
Expression level of biomarkers and Molecular Docking Visualization. **(A)** the gene expression levels of essential genes (BTD, CFL1, PIGR, SERPINA3), **(B)** the protein expression levels of essential proteins (BTD, CFL1, PIGR, SERPINA3). **(C)** The binding between BTD and Paxlovid. **(D)** The binding between CFL1 and Paxlovid. **(E)** The binding between PIGR and Paxlovid. **(F)** The binding between SERPINA3 and Paxlovid.

To determine whether BTD, CFL1, PIGR and SERPINA3 can serve as biomarkers for COVID-19, we verified with the related biomarkers expression of transcriptomics data (**Figure 7A**) and proteomics data (**Figure 7B**). In order to determine whether Paxlovid plays a therapeutic role through biomarkers, we conducted molecular docking verification based on AlphaFold. The results indicated that Paxlovid may bind to BTD (**Figure 7C**), CFL1 (**Figure 7D**), PIGR (**Figure 7E**), and SERPINA3 (**Figure 7F**) with favorable binding energies (**Table 2**), potentially modulating their protein functions and exerting therapeutic effects.

**Table 2:** Molecular docking based on AlphaFold.

Protein	AlphaFold structure ID	Vina score	Center (x, y, z)
Biotinidase	AF-P43251-F1-v4	-7.3	13, 2, -33
Cofilin-1	AF-P23528-F1-v4	-7.5	13, 2, -33
Polymeric immunoglobulin receptor	AF-P01833-F1-v4	-8.4	-15, 6, -10
Alpha-1-antichymotrypsin	AF-P01011-F1-v4	-7.7	1, 5, -19

## Discussion

The COVID-19 pandemic, caused by the SARS-CoV-2 virus, has profoundly affected the world, with millions of confirmed cases and numerous fatalities (24, 25). The disease presents a wide spectrum of clinical manifestations, ranging from mild symptoms to severe respiratory failure, posing significant challenges for both diagnosis and treatment (26). Recent advances in multi-omics molecular profiling have greatly improved our understanding of the transmission dynamics of respiratory viruses on a global scale (27). Additionally, the substantial overlap in clinical symptoms among various respiratory illnesses continues to hinder accurate diagnosis. To address this challenge, we conducted a comprehensive framework using healthy individuals as a control group, collecting clinical diagnostic data from the peripheral blood of both COVID-19 patients and healthy controls. The omics data (GSE192391, GSE164805) and our clinical cohort come from different sources and time points, and key variables such as age, disease severity and gender can be seen in **Supplementary Table S1**. By integrating scRNA-seq and RNA-seq data with an analysis of the peripheral plasma proteome, we applied machine learning models to successfully identify and predict potential biomarkers associated with CD8+ T cell responses in COVID-19 infection. Our findings suggest that BTD, CFL1, PIGR, and SERPINA3 may serve as promising auxiliary diagnostic and therapeutic biomarkers for COVID-19, offering significant clinical potential.

Several limitations of this study should be acknowledged. First, the cohorts used in our analysis were not fully matched in terms of demographic and clinical characteristics, which may introduce confounding factors and limit the comparability across groups. Second, while we integrated both publicly available external datasets (GSE192391, GSE164805) and our own internal proteomic data to enhance robustness, heterogeneity in sample collection, processing protocols, and sequencing platforms could have affected the consistency of the results. Third, the sample size of our internal cohort remains relatively modest, which may constrain the statistical power and generalizability of the findings. Finally, functional validation of the identified biomarkers was not performed in the present work, and further studies in larger, well-matched, and longitudinal cohorts are needed to verify the diagnostic and preventive potential of the proposed multi-omics framework.

The findings are both clinically relevant and biologically plausible. Recent studies suggest critical roles of T cells in the clearance of SARS-CoV-2 and protection from developing severe COVID-19 (28, 29). In a study, Bilich et al. specifically explored the kinetics of SARS-CoV-2–specific T cell responses in two cohorts of patients up to 6 months after infection (30). The authors found that, whereas antibody responses wane, T cell responses to SARS-CoV-2 antigens remain consistent or increase over time. T cell responses against SARS-CoV-2 likely provide protection against severe COVID-19, but how reinfection affects T cell functionality remains unclear (31). The coronavirus typically induces an excessive immune response, with the overconsume and dysfunction of CD8+ T cells being one of the core mechanisms of immunopathological damage (15). SARS-CoV-2-specific CD8+ T cells in pre-pandemic samples from children, adults, and elderly individuals predominantly displayed a naive phenotype, indicating a lack of previous cross-reactive exposures (32). A subset of CD8+ T cells regulate chronic inflammation in COVID-19 patients by killing pathogenic CD4+ T cells (33). Nevertheless, despite extensive research, the exact role of CD8+ T cells in COVID-19 remains to be determined.

Biotinidase (BTD) plays a role in supporting immune function, and since COVID-19 affects immune responses, there may be an indirect relationship (34). BTD deficiency can impair immune function, and individuals with biotinidase deficiency or biotin deficiency may exhibit altered responses to infections, including viral ones like SARS-CoV-2 (35). While BTD has not been studied specifically in the context of COVID-19, its role in cellular processes and immunity suggests that biotinidase could potentially support immune function during viral infections, though there is no evidence yet to suggest it has a direct effect on COVID-19. Cofilin 1 (CFL1) is involved in cytoskeletal regulation and plays a significant role in immune cell migration, particularly may influence T cells migrate to sites of infection and become activated in inflammatory response (36). Therefore, CFL1 could serve as a potential marker for disease severity in COVID-19, with elevated levels or altered actin dynamics possibly correlating with more severe disease or prolonged inflammation. PIGR is essential for mucosal immunity by facilitating the secretion of dimeric IgA and IgM into mucosal surfaces like the respiratory and gastrointestinal tracts (37, 38). The dysregulation of PIGR activity could impair mucosal immune responses, particularly IgA ability to neutralize SARS-CoV-2, leading to higher viral loads in the upper respiratory tract and potentially more severe disease. SERPINA3 plays an important role in regulating inflammation and protecting tissues from protease-mediated damage (39). The decrease of SERPINA3 level may be related to poor outcome, but it may also be a protective factor to limit excessive tissue damage. Their potential role in acute COVID-19 and long-term COVID makes them potential biomarkers of disease severity and a candidate for therapeutic intervention.

Healthcare has shifted from a responsive model to a proactive, personalized, and preventative approach. The development of multi-omics offers a powerful framework to accurately predict an individual’s disease risk and uncover complex biological interactions that may otherwise remain hidden (40). Swinnerton et al. have developed and prospectively validated a tool to predict the absolute risk of severe COVID-19, incorporating dynamic parameters at both the patient and population levels, which could inform clinical care (41). However, it remains possible that this model may not generalize to individuals who acquired immunity via natural infection or those without exposure to the virus or vaccine. The SARS-CoV-2 pandemic spread rapidly worldwide, resulting in high mortality. Developing enhanced vaccination strategies that effectively protect against both disease and viral transmission is crucial for preparing for future respiratory virus pandemics. Leveraging a multi-omics approach in our research allows us to comprehensively assess the multifactorial immune response, providing deeper insights into how blood biomarkers of COVID-19 modulate immunity. Further clinical and translational studies are essential to refine these findings and bridge the gaps in our understanding.

## Conclusions

Our findings suggest that BTD, CFL1, PIGR, and SERPINA3 may serve as promising auxiliary diagnostic and therapeutic biomarkers for COVID-19, offering significant clinical potential. By combining machine learning with multi-omics framework, we offer a novel approach to precision medicine, especially in early diagnosis and personalized treatment.

## Data Availability

The scRNA-seq dataset can be obtained from GEO with the primary accession code GSE192391, and RNA-Seq data can be obtained from GEO with the primary accession code GSE164805. The proteomics datasets used in this study are available from the corresponding author on reasonable request.

## Supplementary material

Supplementary Table 1 Cohort Alignment and Characterization.
