# Supplementary material for: Multi-omics and machine learning identify novel biomarkers and therapeutic targets of COVID-19
Source: Front Immunol. 2025 Oct 2;16:1671936. doi: 10.3389/fimmu.2025.1671936 (PMC12528157; doi:10.3389/fimmu.2025.1671936)
Supplement: Supplementary Table 1 — Cohort Alignment and Characterization. [file Table1.docx]

**Table S1 Cohort Alignment and Characterization**

| Omics data | GSE192391 | | GSE164805 | | Proteomic data | |
| --- | --- | --- | --- | --- | --- | --- |
|  | Control(n=6) | COVID-19(n=12) | Control(n=24) | COVID-19(n = 27) | Control(n=265) | COVID-19(n=358) |
| Age(year) | 70+/-8.6 | 70+/-11.5 | 48.0 (25-76) | 50.0 (13-70) | 60(15-97) | 68(7-91) |
| Gender(male%) | 33 | 75 | 58 | 67 | 55 | 58 |
| Ethnicity(white%) | 50 | 33 | NA | NA | NA | NA |
| Disease severity | NA | Severe | NA | Severe | NA | Severe |
